# Hypomethylation-associated ELF3 helps nasopharyngeal carcinoma to escape immune surveillance via MUC16-mediated glycolytic metabolic reprogramming

**DOI:** 10.1152/ajpcell.00438.2024

**Published:** 2024-09-02

**Authors:** Yueyang Liu, Hong Zhou, Qi Yu, Qiang Wang

**Affiliations:** ^1^Otolaryngology & Head and Neck Center, Cancer Center, Department of Otolaryngology, Zhejiang Provincial People’s Hospital (Affiliated People’s Hospital), Hangzhou Medical College, Hangzhou, People’s Republic of China; ^2^Center for Rehabilitation Medicine, Department of Orthopedics, Zhejiang Provincial People’s Hospital (Affiliated People’s Hospital), Hangzhou Medical College, Hangzhou, People’s Republic of China

**Keywords:** ELF3, glycolysis, immune escape, MUC16, nasopharyngeal carcinoma

## Abstract

Immune escape and metabolic reprogramming are two essential hallmarks of cancer. Mucin-16 (MUC16) has been linked to glycolysis and immune response in different cancers. However, its involvement in nasopharyngeal carcinoma (NPC) has not been well described. We seek to dissect the functions and detailed mechanisms of MUC16 in NPC. Bioinformatics prediction was performed to identify NPC-related molecules. MUC16 was significantly enhanced in NPC tissues, which was correlated with the advanced tumor stage of patients. Lentiviral plasmids-mediated MUC16 deletion inhibited the malignant behavior of NPC cells, and glycolysis inhibition by MUC16 deletion blocked immune escape in NPC cells. E74-like factor 3 (ELF3) bound to the MUC16 promoter promotes the transcription of MUC16. MUC16 overexpression reversed the repressive effect of ELF3 silencing on glycolysis and immune escape in NPC and accelerated tumor growth in vivo. Overexpression of ELF3 in NPC was associated with reduced DNA methylation in its promoter. Our findings revealed the role of the ELF3/MUC16 axis in the immune escape and metabolic reprogramming of NPC, providing potential therapeutic targets for NPC.

**NEW & NOTEWORTHY** We identified the functions of E74-like factor 3 (ELF3) in glycolysis and immune escape of nasopharyngeal carcinoma cells for the first time. As a transcription factor, ELF3 promoted mucin-16 (MUC16) expression by binding to its promoter, leading to the glycolysis-mediated immune escape of nasopharyngeal carcinoma (NPC) cells. Targeting the ELF3/MUC16 axis generates a superior antitumor immune response, which will help establish a novel approach to restore protective antitumor immunity for NPC immunotherapy.

## Graphical abstract

**Figure:**
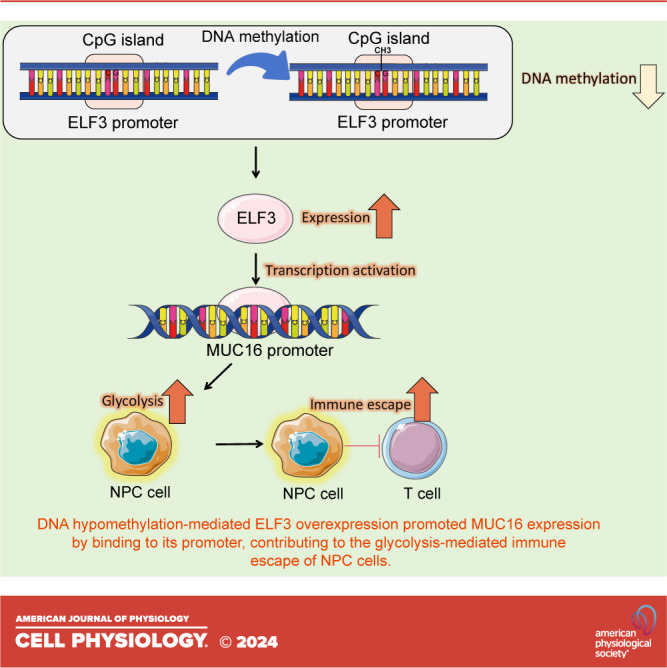

## INTRODUCTION

Nasopharyngeal carcinoma (NPC) is particularly prevalent in East and Southeast Asia, while mortality has been reduced substantially (1). Despite a favorable prognosis with the 5-yr survival rate for combined chemoradiotherapy reaching 85–90%, around 8–10% of patients experience recurrence or metastasis after treatment regimens (2). Therefore, developing novel therapeutic strategies to improve the outcomes of NPC is urgently needed.

To deal with the demand for macromolecules and bioenergy, tumor cells reconfigure their metabolic flux to accelerate their initiation, growth, and metastatic abilities (3). In detail, normal cells obtain their energy from oxidative phosphorylation, whereas cancer cells obtain their energy from oxidative glycolysis, known as the “Warburg effect” (4). The hallmark of cancer is extracellular acidification caused by lactate accumulation, and lactate is an essential metabolite in the tumor microenvironment (TME) as it plays a crucial part in shaping immune cell function and inhibits the activation and proliferation of immune cells (5). The coexistence of Epstein–Barr virus-infected NPC cells and tumor-infiltrating lymphocytes represents a highly heterogeneous and suppressive TME that fuels immune escape and promotes tumorigenesis (6). However, the specific mechanism responsible for glycolysis-related immune escape in NPC has not been clarified.

The Mucin (MUC) family, highly glycosylated macromolecules, is ubiquitously expressed in mammalian epithelial cells and pivotal in establishing protective mucosal barriers, serving as defenses against pathogenic assaults (7). Mucin-16 (MUC16) is a membrane-bound mucin that is abnormally expressed or mutated in a variety of diseases, especially tumors, and can be regarded as an important cancer-related biomarker (8). In this study, MUC16 was identified among the most significantly differentially expressed genes (DEGs) in NPC relative to counterparts. MUC16 (also termed CA125) has been used as a biomarker for ovarian cancer, and its expression is related to disease progression (9). Interestingly, MUC16 has been linked to glucose uptake and glycolysis in gallbladder carcinoma by binding and enhancing the protein stability of fructose-bisphosphate aldolase C (10). In addition, variable MUC16 can help ovarian cancer cells avoid immune cytotoxicity and form metastases (11). However, its function in NPC has not been established. Besides MUC16, E74-like factor 3 (ELF3) was the only transcription factor that is also differentially expressed in NPC in our included Gene Expression Omnibus (GEO) datasets. ELF3, located on chromosome 1q32.1 and encodes a 371 aa protein, is a member of the epithelium-specific E26 transformation-specific transcription factors and has been related to bladder, cervical, ovarian, and gastrointestinal cancers (12). Circular homeodomain interacting protein kinase 3 has been reported to facilitate NPC development by protecting ELF3 from microRNA-4288-mediated silencing (13). ELF3 was also among the most upregulated genes in more advanced lung adenocarcinoma cells and correlated to heterogeneous tumor and immune cell populations (14). Here, we aimed to expound the mechanism of MUC16 in NPC cell glycolysis and tumor progression and to determine the upstream mechanism, thus leading to tumor development.

## MATERIALS AND METHODS

### Patient Sample Collection

A total of 23 tumor biopsy tissue samples and paired paracancerous nasopharyngeal tissue samples were harvested from March 2021 to May 2023 at Zhejiang Provincial People’s Hospital. All patients with NPC did not receive any chemotherapy or radiotherapy before the biopsy. All tissue samples were made into paraffin sections and stored in a −80°C refrigerator. The Institutional Research Ethics Committee of Zhejiang Provincial People’s Hospital approved this study, and this study was performed according to the Declaration of Helsinki. Informed consent was obtained from all subjects.

### Cell Culture

NPC cells C666-1 (JY507) and HK-1 (JY508) were purchased from SSRCC (Shanghai, PR China). Both were grown in RPMI-1640 medium plus 10% FBS. Nasopharyngeal epithelial cells NP-69 (CL-0804) were purchased from Procell (Wuhan, Hubei, PR China) and cultured in NP69 cell-specific medium (CM-0804, Procell). Peripheral blood mononuclear cells (PBMC) (PCS-800-011) were acquired from the ATCC (Manassas, VA), and we isolated CD8^+^ T cells from PBMC using the Dynabeads Untouched Human CD8 T Cell Kit (11348D, Thermo Fisher Scientific, Inc., Waltham, MA). CD8^+^ T cells were stimulated with Dynabeads Human T Activator CD3/CD28 (11161D, Thermo Fisher Scientific, Inc.) and grown in RPMI-1640 medium in 10% FBS. All cell culture environments were 37°C with 5% CO_2_. For inhibition of glycolysis and DNA methylation, NPC cells were treated with 50 μM 2-deoxy-d-glucose (2-DG) (HY-13966, MedChemExpress, Monmouth Junction, NJ) for 24 h or 20 μM 5-azacytidine (HY-10586, MedChemExpress) for 96 h, respectively. DMSO was used for control treatment.

### Lentiviral Infection

The lentiviral plasmids used to knockdown MUC16 (pLV[shRNA]-EGFP:T2A: Puro-U6>hMUC16[shRNA]), knockdown ELF3 (pLV[shRNA]-EGFP:T2A: Puro-U6>hELF3[shRNA]), or overexpress MUC16 (pLV[Exp]-mCherry:T2A: Puro-EF1A>hMUC16 [NM_001414686.1]) were designed by VectorBuilder (Guangzhou, Guangdong, PR China). C666-1 and HK-1 cells were plated into 6-well plates and cultured for 24 h. After the medium was discarded, 1 mL of enhanced infection solution containing 5 μg/mL Polybrene (HY-112735, MedChemExpress) was added. Cells were infected with lentiviral vectors containing the target gene and control at multiplicity of infection (MOI) of 30, respectively. The culture medium was refreshed after 24 h of infection, and the infection efficiency was assessed by observing the fluorescence using an inverted fluorescence microscope. The following shRNAs were generated: hMUC16 [shRNA]: AATGTCACAGACCAATAGAGACACGTTTAATGACTCTGCTG; hELF3[shRNA]: TCCAGAGTCGGAACTGAGGGTTGGAACTATACCCGGGACCA.

### Ethynyl Deoxyuridine Incorporation Assay

NPC cells with knockdown of MUC16 were collected, and an ethynyl deoxyuridine (EdU) Cell Proliferation Assay Kit (E607204, Sangon Biotech, Shanghai, PR China) was used. NPC cells were treated with an EdU-labeled medium for 2 h and fixed with 4% paraformaldehyde for 0.5 h. Glycine was added to neutralize the excess formaldehyde. The cells were permeabilized using 0.5% Triton X-100 for 10 min, reacted with staining reaction solution containing 6-carboxy-tetramethyl rhodamine red fluorescent solution, and washed using 0.5% Triton X-100 permeabilizing solution. The nuclei were counterstained with DAPI at room temperature, and the cells were viewed under a fluorescence microscope. The rate of EdU^+^ cells (%) was assessed.

### Colony Formation Assay

Transfected NPC cells were detached into single cells and cultured for 2 wk in 96-well plates at 37°C, 5% CO_2_. After that, the cells were fixed with 4% paraformaldehyde solution for 10 min and stained with 0.1% crystal violet for 15 min. The colony-forming ability was assessed by counting the number of colonies.

### Wound Healing Assay

NPC cells were collected and grew to a confluent monolayer. The medium was discarded, and a pipette tip was used to create scratches. The cells were incubated with a serum-free culture medium. Using images captured at 0 and 24 h, the width of the scratch was determined using ImageJ.

### Matrigel Invasion Assay

Transwell chambers (8 µm, Corning Glass Works, Corning, NY) were used. NPC cells (10^5^) were washed and spread into the apical chamber that was precoated with Matrigel, and the basolateral chamber was added with fresh medium containing 10% FBS. After being incubated for 24 h in a cell culture incubator, the invaded cells in the basolateral chamber were fixed, stained with crystal violet, and photographed under a microscope. The number of invaded cells was used as a measure of invasion capacity.

### Extracellular Acidification Rate

A Seahorse XFe96 instrument (Seahorse Bioscience, North Billerica, MA) was used. The cells were loaded into Seahorse 96-well plates at 7,500 cells/well, equilibrated with XF basal medium for 1 h at 37°C in a CO_2_-free incubator, and starved for 60 min in a glucose-free medium. The cells were treated sequentially with glucose, oligomycin, and 2-DG as described in the XF Glycolytic Stress Test Protocol using the Seahorse XFe96 Extracellular Flux Analyzer. The extracellular acidification rate (ECAR; mpH/min) was assessed using the Glycolytic Stress Test Kit.

### Glucose Uptake and Lactate Production Assessment

NPC cells were treated with the glucose-free medium at 37°C with 5% CO_2_ for 1 h and with a glucose-free medium containing 50 μM 2-[*N*-(7-nitrobenz-2-oxa-1,3-diazol-4-yl) amino]-2-deoxy-d-glucose (2-NBDG) (HY-116215, MedChemExpress) for 30 min, and glucose uptake was assessed by fluorescence imaging microscopic observation and calculation of fluorescence intensity.

Lactate Content Assay Kit (D799099, Sangon Biotech) was also used. Briefly, C666-1 and HK-1 cells were cultured for 24 h, followed by ultrasonication on ice with the addition of *extract I*. After a 12,000 *g* centrifugation for 10 min at 4°C, the supernatant was harvested for another centrifugation under the same condition with the addition of *extract II*. The supernatant was subsequently added with the assay components, and the reaction was carried out in a water bath at 37°C in the dark. Centrifugation was performed, and the precipitate was dissolved by ethanol. The optical density (OD) value was read at 570 nm to assess the lactate production.

### Coculture of T Cells with NPC Cells

To evaluate the immune escape of NPC cells, we constructed a coculture system using Transwell chambers. We added T cells and NPC cells into the apical and basolateral chambers at the effector-to-target ratio of 10:1 (15) and transferred the chambers to the incubator at 37°C with 5% CO_2_ for 12 h. Cells from the apical and basolateral chambers and the coculture supernatant were collected separately at the end of the culture for subsequent analysis.

### Flow Cytometry

Annexin V-FITC/PI Apoptosis Detection Kit (HY-K1073, MedChemExpress) was used to evaluate NPC cell apoptosis. The cells were resuspended with binding buffer and incubated with 5 µL Annexin V-FITC and 10 µL propidium iodide (PI) staining solution at room temperature for 15 min in darkness, and loaded into flow cytometry.

For the detection of effector activation in T cells, T cells were harvested after coculture, resuspended in binding buffer, and probed with antibodies to Perforin-APC (17-9994-42, Thermo Fisher Scientific, Inc.) and Granzyme B-APC (GRB05, Thermo Fisher Scientific, Inc.) for 0.5 h at 4°C in darkness. The percentage of perforin- and granzyme B^+^ cells in T cells was measured in a flow cytometer.

### ELISA

Human tumor necrosis factor α (TNF-α) ELISA Kit (E-EL-H0109, Elabscience, Wuhan, Hubei, PR China), human interferon-γ (IFN-γ) ELISA Kit (E-EL-H0108, Elabscience), mouse TNF-α ELISA Kit (E-EL-M3063, Elabscience), and mouse IFN-γ ELISA Kit (E-EL-M0048, Elabscience) were applied for the detection of TNF-α and IFN-γ in coculture supernatants and xenograft tumor tissues.

### Dual-Luciferase Assay

The MUC16 promoter was cloned into the pGL3-Basic (E1751, Promega Corporation, Madison, WI) firefly luciferase (F-Luc) reporter plasmid to generate a luciferase reporter plasmid. The plasmids were transfected into C666-1 or HK-1 cells with ELF3 knockdown for 48 h. Firefly luciferase (F-Luc) and Renilla luciferase (R-Luc) activities were assessed using the Dual-Glo Dual Fluorokinase Reporter Gene Assay System (E2920, Promega).

### Chromatin Immunoprecipitation

Chromatin immunoprecipitation (ChIP) was conducted to analyze the binding relationship between ELF3 and the MUC16 promoter. Briefly, C666-1 and HK-1 cells were treated with formaldehyde to crosslink chromatin-associated proteins and DNA, and chromatin was extracted by ultrasonic fragmentation and probed with IgG (1:50, No. 3900, Cell Signaling Technologies, Beverly, MA) and anti-ELF3 (1:50, MA5-35683, Thermo Fisher Scientific, Inc.) at 4°C overnight. Protein G agarose magnetic beads were used to separate the immunoprecipitation complexes. The agarose complexes were resuspended and centrifuged to harvest the protein/DNA complexes. The DNA-protein cross-links were eluted. The obtained DNA was purified, followed by qPCR to assess the relative occupancy of the MUC1 promoter fragment in the DNA fragments (normalized to IgG).

### Xenograft Assay

The Institutional Animal Care and Use Committee of Zhejiang Provincial People’s Hospital approved all animal experiments. Six-week male Balb/c mice with normal immunoreactivity were purchased from Vital River (Beijing, PR China) and acclimatized under specific pathogen-free conditions for 2 wk. The mice were randomized into four groups: the lentiviruses (LV)-negative control (NC)-knockdown (KD), LV-ELF3-KD, LV-ELF3-KD + NC-overexpression (OE), and LV-ELF3-KD + MUC16-OE groups (*n* = 8). Cell suspensions containing 1 × 10^7^ C666-1 cells were subcutaneously administrated into the back of each mouse according to grouping. Tumor size was assessed at an interval of 7 days using vernier calipers, and tumor volume was calculated as length × width^2^ × 0.5. After the last measurement of tumor volume on the 28th day, the mice were euthanized with 150 mg/kg pentobarbital sodium (ip), and tumor tissues were weighed and paraffin-embedded.

### Immunohistochemistry

NPC tissue and subcutaneous xenografts were dewaxed by xylene and rehydrated with gradient ethanol. After microwave heating for antigen retrieval, the sections were incubated in 3% hydrogen peroxide solution for 10 min and sealed with goat serum for 2 h. The sections were probed with antibodies to MUC16 (1:100, GTX03800, GeneTex, Inc., Alton Pkwy, Irvine, CA), ELF3 (1:100, PA5-120996, Thermo Fisher Scientific, Inc.), glucose transporter 1 (GLUT1, 1:100, A6982, ABclonal, Wuhan, Hubei, PR China), LDHA (1:200, PA5-27406, Thermo Fisher Scientific, Inc.), and CD8 (1:100, A11856, ABclonal) overnight at 4°C and with horseradish peroxidase (HRP)-coupled secondary antibody (1:1,000, AS029, ABclonal) for 2 h. Diaminobenzidine (DAB) substrate was added to visualize the staining. After washing was completed, the sections were stained with hematoxylin, sealed with neutral gum, and viewed under a microscope.

### Methylation-Specific PCR

Genomic DNA was extracted from NPC samples and cells and modified with sodium bisulfite. DNA samples were first extracted using the PureLink Genomic DNA Mini-Extraction Kit (K182000, Thermo Fisher Scientific, Inc.), and bisulfite conversion was performed using the EpiTect Bisulfite Kit (59104, Qiagen company, Hilden, Germany). qPCR or agarose gel separation was performed based on primers specific for either methylated or unmethylated ELF3 (designed by Sangon Biotech). The sequences of the methylated-specific (M) primer and unmethylated-specific (U) primers were the following: left M primer, TTGTAAGAAGGGGGATTTTAAGTAC and right M primer, ATACGAAAACCAAAAAAAACGAA; left U primer, TTGTAAGAAGGGGGATTTTAAGTAT and right U primer, ACCATACAAAAACCAAAAAAAACAA.

### Reverse Transcription-Quantitative Polymerase Chain Reaction

Total RNA was extracted from tumor tissues and NPC cells using the TRIzol Kit. First-strand cDNA was obtained by reverse transcription reaction using RT Master Mix for qPCR (gDNA digester plus) (HY-K0511, MedChemExpress), followed by qPCR using SYBR Green qPCR Master Mix (HY-K0501, MedChemExpress). Gene levels were normalized to β-actin and calculated using the 2^−ΔΔCt^ method. The primers were as follows: *MUC16*, 5′-GATGTCAAGCCAGGCAGCACAA-3′ (forward) and 5′-GAGAGTGGTAGACATTTCTGGGC-3′ (reverse); *ELF3*, 5′-CATGACCTACGAGAAGCTGAGC-3′ (forward) and 5′-GACTCTGGAGAACCTCTTCCTC-3′ (reverse); β-actin 5′-CACCATTGGCAATGAGCGGTTC-3′ (forward) and 5′-AGGTCTTTGCGGATGTCCACGT-3′ (reverse).

### Western Blotting

RIPA buffer was used to extract total protein from NPC cells, which was quantified using the bicinchoninic acid (BCA) method. The proteins were separated by SDS-PAGE and transferred to a PVDF membrane. The membrane was sealed using 5% skim milk powder in phosphate-buffered saline for 2 h and probed with primary antibodies to MUC16 (1:500, A4666, ABclonal), GLUT1 (1:1,000, A6982, ABclonal), LDHA (1:1,000, No. 2012, Cell Signaling Technologies), PKM2 (1:1,000, No. 4053, Cell Signaling Technologies), and β-actin (1:50,000, AC026, ABclonal) at 4°C overnight. Next, the membranes were re-probed with HRP-coupled secondary antibody (1:5,000, AS029, ABclonal) for 2 h at room temperature. The blots were developed using the ECL substrate kit and analyzed using ImageJ.

### Statistics and Reproducibility

All statistical analyses were performed using the GraphPad Prism 8.0.2 software (GraphPad, San Diego, CA). All results are presented as the means ± SD. Association between genes was assessed by Pearson’s correlation analysis, and the correlation between clinical characteristics and gene expression was calculated using the χ^2^ test or Fisher’s exact test. Statistical significance was calculated using a paired *t* test or one-way/two-way ANOVA. *P* < 0.05 was assigned as the statistical significance.

## RESULTS

### MUC16 Is Elevated in NPC Tissues and Correlated with Clinicopathological Features of Patients

**Figure 1.**
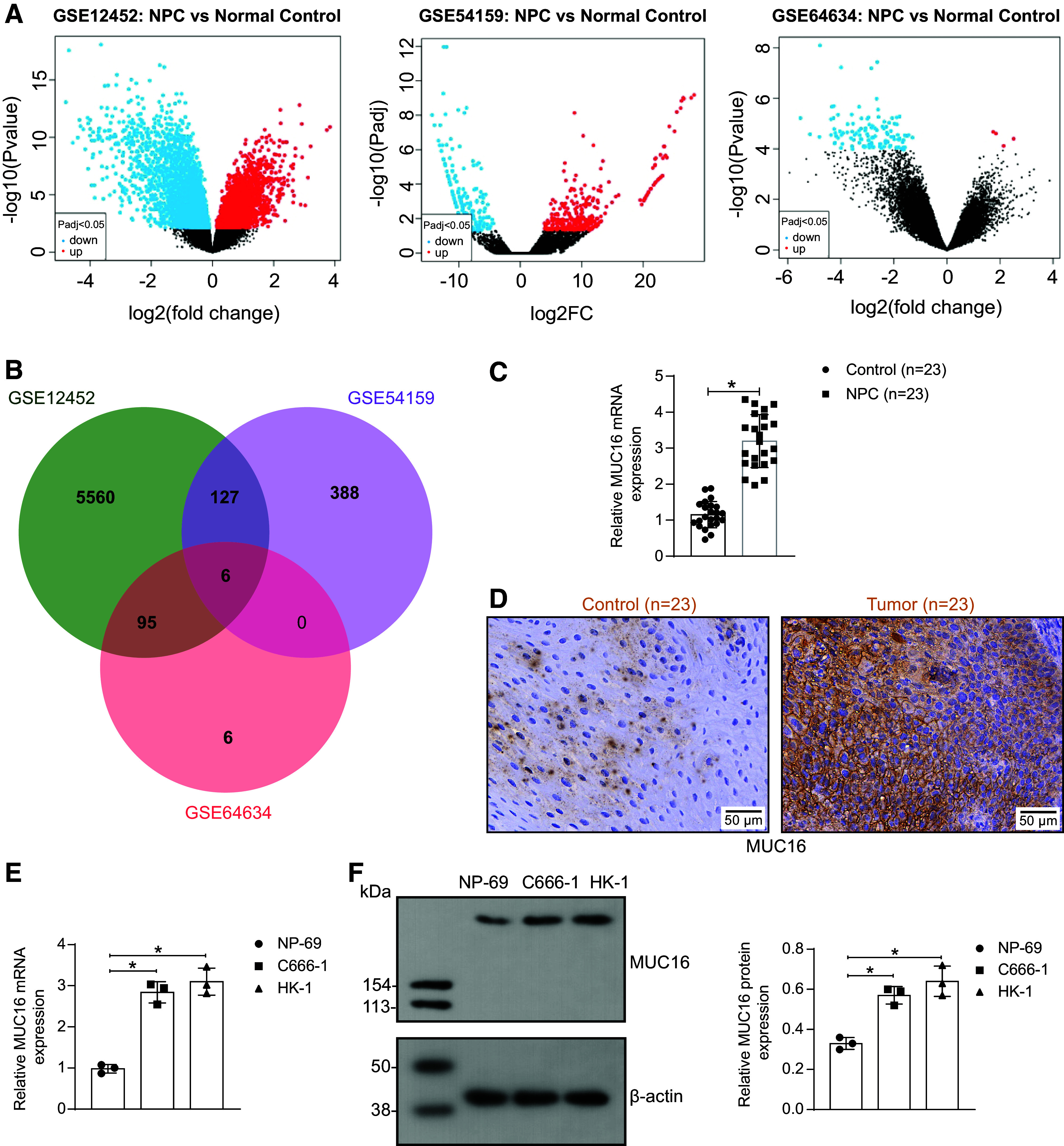
Mucin-16 (MUC16) is overexpressed in nasopharyngeal carcinoma (NPC) and correlates with clinicopathological features of patients. *A*: volcano plots in NPC samples and normal control samples in the GSE12452, GSE54159, and GSE64634 datasets (Benjamini and Hochberg corrected for *P* value, threshold at *P* < 0.05). *B*: Venn map of differentially expressed genes (DEGs) in the GSE12452, GSE54159, and GSE64634 datasets. *C*: *MUC16* mRNA expression in NPC samples and normal adjacent samples was analyzed using RT-qPCR (*n* = 23 patients). *D*: representative immunohistochemical staining images of MUC16 in NPC samples and normal adjacent samples. *E*: *MUC16* mRNA expression in nasopharyngeal epithelial cells NP-69 and NPC cells (C666-1 and HK-1 cells) were analyzed using RT-qPCR (*n* = 3). *F*: the protein expression of MUC16 in nasopharyngeal epithelial cells NP-69 and NPC cells (C666-1 and HK-1 cells) was analyzed using Western blotting (*n* = 3). Data are shown as mean values ± SD from three independent experiments. RT-qPCR, reverse transcription-quantitative polymerase chain reaction. *P < 0.05 (*t* test for *C* or ANOVA for *E* and *F*).

We analyzed the transcriptome differences in NPC samples and normal control samples by GEO datasets GSE12452 (total RNA extracted from laser-captured epithelium from 31 NPC and 10 normal healthy nasopharyngeal tissues), GSE54159 (matched total mRNA of undifferentiated NPC xenograft X666 and its derived cell line C666, well-differentiated NPC cell line HK1, and the immortalized nasopharyngeal epithelial cell line NP460), and GSE64634 (total RNA extracted from laser-captured epithelium from 12 NPC and 4 normal healthy nasopharyngeal tissue specimens) using the GEO2R tool. DEGs were filtered by Benjamini and Hochberg correction of *P* value at *P* < 0.05 (Fig. 1*A*). There were a total of six intersecting DEGs in the three databases (Fig. 1*B*): *MUC16*, *SLC44A4*, *PRR15*, *TSPAN1*, *WFDC2*, and *ELF3*. Compared with the other five genes, *MUC16* was the most significant in two of the three GEO datasets (*P* = 0.000261 in GSE54159 and *P* = 1.67*e*-11 in GSE12452).

In our NPC cohort, we found a remarkable increase in the expression of *MUC16* (Fig. 1, *C* and *D*). We then categorized the patients into *MUC16* high-expression and low-expression groups according to the average mRNA expression of *MUC16*. As shown in Table 1, there was a significant correlation between *MUC16* and tumor, node, metastases (TNM) stage and recurrence (*P* < 0.05), but not with patients’ sex, age, and smoking history (*P* > 0.05). In nasopharyngeal epithelial cells and NPC cells, we also found increased expression of *MUC16* in NPC cell lines (Fig. 1, *E* and *F*), suggesting that the expression of *MUC16* in NPC may be directly related to the malignant progression.

**Table 1.** Association between MUC16 level and clinicopathological characteristics in patients with NPC

Variables	NPC (*n* = 23)	Low MUC 16 Expression (*n* = 12)	High MUC 16 Expression (*n* = 11)	*P* Value
Sex				
Female	6	4	2	ns
Male	17	8	9	
Years				
≥50	13	9	4	ns
<50	10	3	7	
Smoking				
Yes	15	7	8	ns
No	8	5	3	
TNM				
≥III	10	2	8	0.0123*
<III	13	10	3	
Recurrence				
Yes	9	2	7	0.0361*
No	14	10	4	

MUC16, mucin-16; NPC, nasopharyngeal carcinoma; ns, not significant; TNM, tumor, node, metastases. *P < 0.05.

### Knockdown of *MUC16* Inhibits the Malignant Behavior of NPC Cells

**Figure 2.**
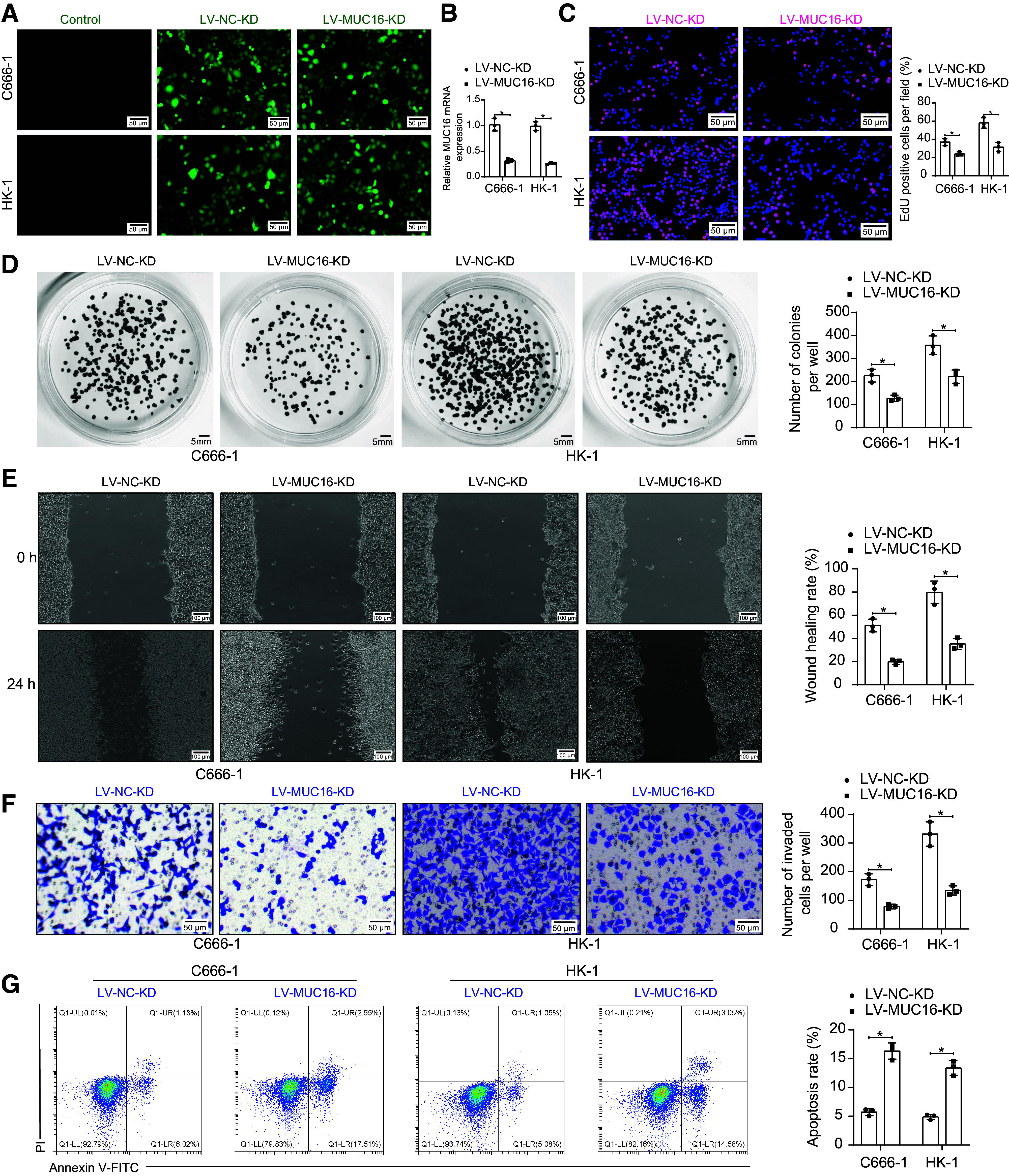
Knockdown of mucin-16 (MUC16) inhibits malignant behavior of nasopharyngeal carcinoma (NPC) cells. *A*: infection efficiency of lentiviral vectors infecting C666-1 and HK-1 cells by fluorescence microscopy (*n* = 3). *B*: MUC16 mRNA expression in C666-1 and HK-1 cells infected with lentiviral vectors harboring MUC16 knockdown was analyzed using RT-qPCR (*n* = 3). *C*: the proliferative capacity of C666-1 and HK-1 cells infected with lentiviral vectors harboring MUC16 knockdown was analyzed using ethynyl deoxyuridine (EdU) staining (*n* = 3). *D*: the colony-forming ability of C666-1 and HK-1 cells infected with lentiviral vectors harboring MUC16 knockdown was analyzed using colony formation assay (*n* = 3). *E*: the migration of C666-1 and HK-1 cells infected with lentiviral vectors harboring MUC16 knockdown was evaluated using a wound healing assay (*n* = 3). *F*: the invasive ability of C666-1 and HK-1 cells infected with lentiviral vectors harboring MUC16 knockdown was evaluated using Matrigel invasion assay (*n* = 3). *G*: apoptosis levels in C666-1 and HK-1 cells infected with lentiviral vectors harboring MUC16 knockdown were evaluated using flow cytometry (*n* = 3). Data are shown as mean values ± SD from three independent experiments. KD, knockdown; NC, negative control; RT-qPCR, reverse transcription-quantitative polymerase chain reaction. *P < 0.05 (ANOVA).

MUC16 expression was inhibited in C666-1 and HK-1 using lentiviral vectors. After verifying the efficacy of knockdown (Fig. 2, *A* and *B*), we first analyzed the cell proliferation, and knockdown of MUC16 inhibited the proliferation of NPC cells (Fig. 2*C*). Colony formation assays also confirmed that deletion of MUC16 resulted in a greatly reduced colony-forming capacity of the cells (Fig. 2*D*). The reduction of MUC16 repressed NPC cell migration and invasion in wound assay and Matrigel invasion assay (Fig. 2, *E* and *F*). Finally, we analyzed the level of apoptosis, and flow cytometry showed that apoptosis was induced by the deletion of MUC-1 in C666-1 and HK-1 cells (Fig. 2*G*).

### Knockdown of *MUC16* Inhibits Glycolysis in NPC Cells

**Figure 3.**
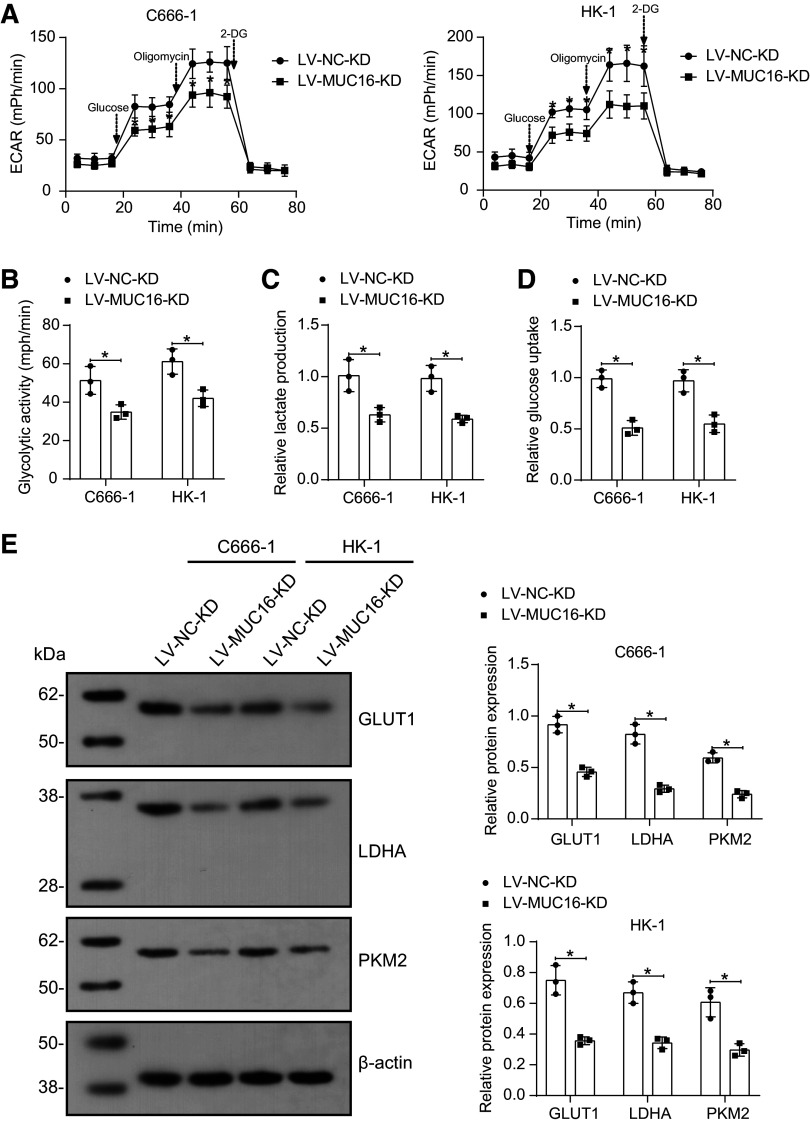
Knockdown of mucin-16 (MUC16) inhibits glycolysis in nasopharyngeal carcinoma (NPC) cells. *A*: assessment of extracellular acidification rate (ECAR) in C666-1 and HK-1 cells infected with lentiviral vectors harboring MUC16 knockdown (*n* = 3). *B*: glycolytic activity in C666-1 and HK-1 cells infected with lentiviral vectors harboring MUC16 knockdown (*n* = 3). *C*: glucose uptake capacity of C666-1 and HK-1 cells infected with lentiviral vectors harboring MUC16 knockdown (*n* = 3). *D*: lactate production of C666-1 and HK-1 cells infected with lentiviral vectors harboring MUC16 knockdown (*n* = 3). *E*: the protein expression of GLUT1, LDHA, and PKM2 in C666-1 and HK-1 cells infected with lentiviral vectors harboring MUC16 knockdown was analyzed using Western blotting (*n* = 3). Data are shown as mean values ± SD from three independent experiments. GLUT1, glucose transporter 1; KD, knockdown; NC, negative control. *P < 0.05 (ANOVA).

First, we analyzed the glycolytic flux, and knockdown of MUC16 contributed to a significant reduction in ECAR levels (Fig. 3, *A* and *B*). We also found that the knockdown of MUC16 reduced the cellular uptake of glucose and decreased lactate production (Fig. 3, *C* and *D*). The expression of glycolysis-related proteins GLUT1, LDHA, and PKM2 in cells showed that the knockdown of MUC16 downregulated glycolytic enzymes (Fig. 3*E*), suggesting that MUC16 can inhibit cellular glycolysis.

### Knockdown of *MUC16* Inhibits Glycolysis in NPC Cells and Blocks Immune Escape

**Figure 4.**
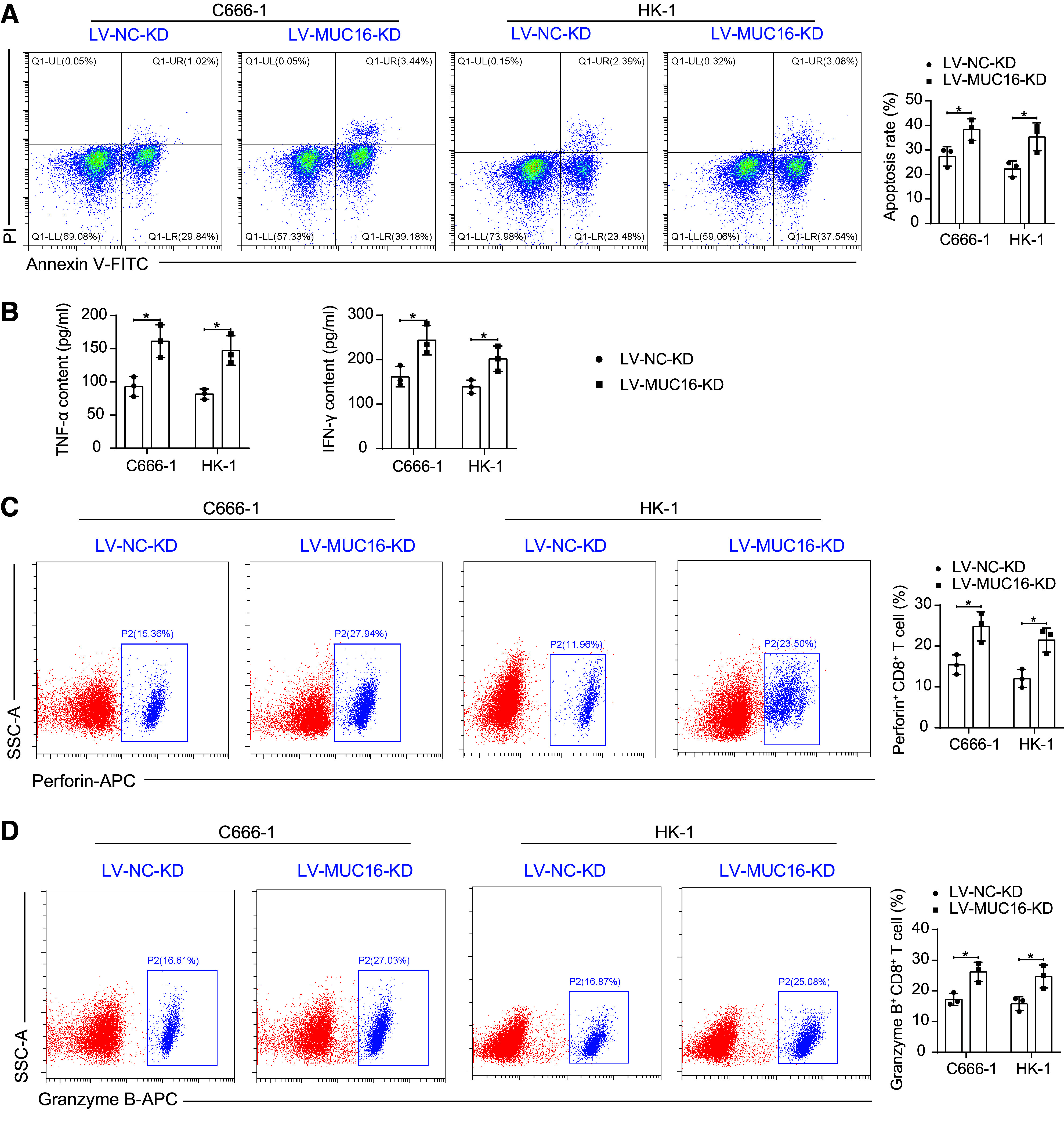
Knockdown of mucin-16 (MUC16) inhibits glycolysis in nasopharyngeal carcinoma (NPC) cells to block immune escape. *A*: detection of apoptosis in C666-1 and HK-1 cells after coculture of T cells with NPC cells with MUC16 knockdown by flow cytometry (*n* = 3). *B*: detection of TNF-α and IFN-γ in the coculture system of T cells and C666-1 and HK-1 cells with MUC16 knockdown analyzed using ELISA (*n* = 3). Percentage of perforin^+^CD8^+^ T cells (*C*) and Granzyme B^+^CD8^+^ T cells (*D*) in T cells after coculture with C666-1 and HK-1 cells with MUC16 knockdown detected by flow cytometry (*n* = 3). Data are shown as mean values ± SD from three independent experiments. KD, knockdown; NC, negative control. *P < 0.05 (ANOVA).

We first cocultured NPC cells knocked down with MUC16 with T cells and analyzed the apoptosis of NPC cells. An increase in apoptosis of NPC cells was observed after knocking down MUC16 (Fig. 4*A*). Further analysis of effector activation of T cells after coculture showed that deletion of MUC16 in NPC cells in the coculture system contributed to promotion in IFN-γ and TNF-α secretion by T cells (Fig. 4*B*). The percentage of perforin^+^CD8^+^ T cells and granzyme B^+^CD8^+^ T cells were analyzed by flow cytometry. MUC16 deletion induced an increase in the proportion of perforin^+^ and granzyme B^+^ cells in the T cells (Fig. 4, *C* and *D*), suggesting that the presence of MUC16 is associated with the dysfunction of T cells in the tumor microenvironment of NPC.

**Figure 5.**
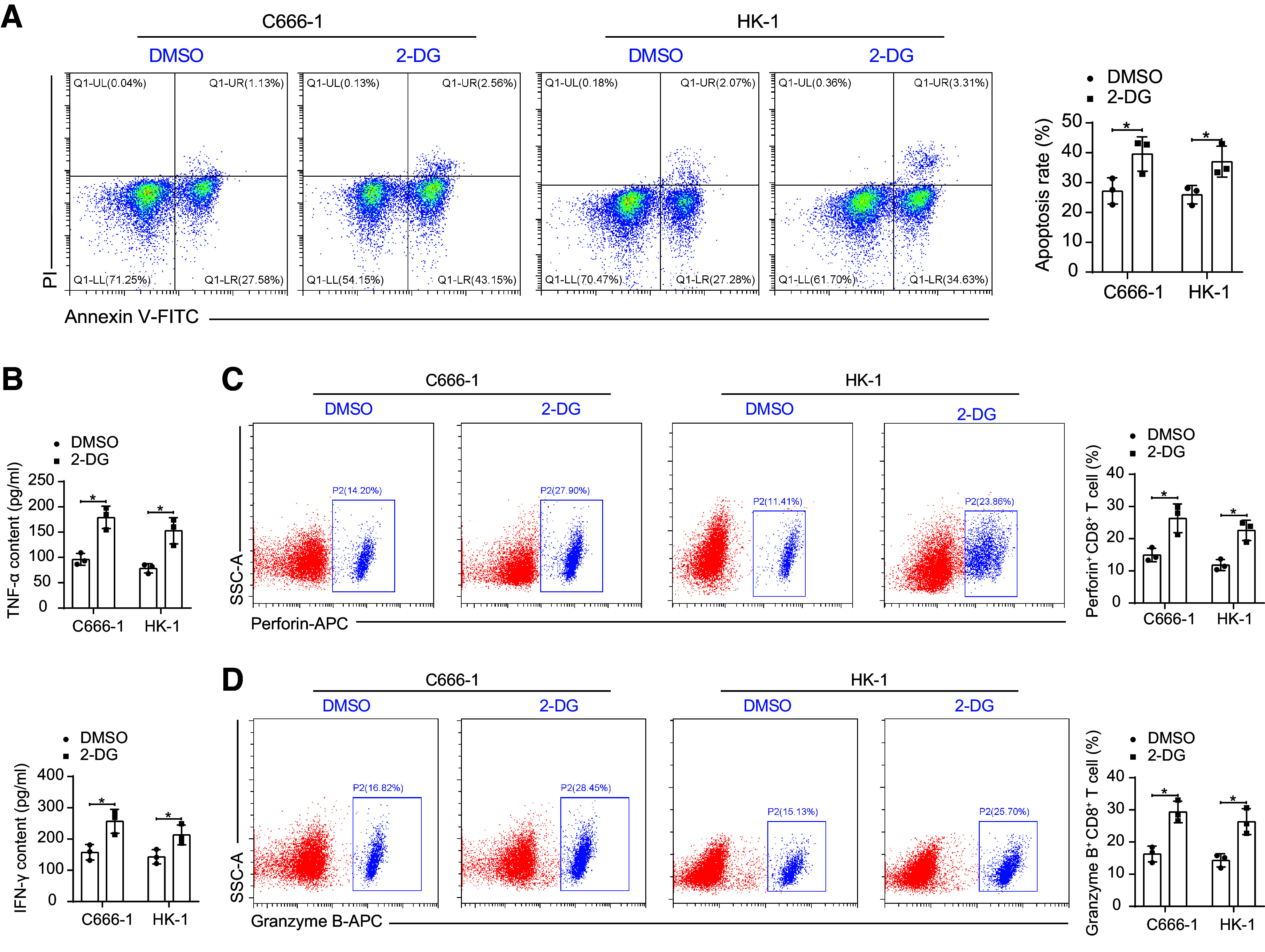
Inhibition of glycolysis promotes immune escape of nasopharyngeal carcinoma (NPC) cells. *A*: detection of apoptosis in C666-1 and HK-1 cells after coculture of T cells with glycolysis inhibitor 2-deoxy-d-glucose (2-DG)-treated C666-1 and HK-1 cells by flow cytometry (*n* = 3). *B*: TNF-α and IFN-γ in a coculture system of T cells and glycolysis inhibitor 2-DG-treated C666-1 and HK-1 cells analyzed using ELISA (*n* = 3). Percentage of perforin^+^CD8^+^ T cells (*C*) and Granzyme B^+^CD8^+^ T cells (*D*) in T cells after coculture with C666-1 and HK-1 cells with MUC16 knockdown detected by flow cytometry (*n* = 3). Data are shown as mean values ± SD from three independent experiments. MUC16, mucin-16. *P < 0.05 (ANOVA).

To substantiate whether MUC16-mediated immune escape is related to glycolysis, we treated NPC cells with the glycolysis inhibitor 2-DG and again analyzed the effector activation of T cells in the coculture system. Inhibition of glycolysis in NPC cells induced effector activation of T cells, as manifested by significantly increased apoptosis of NPC cells (Fig. 5*A*), promoted contents of IFN-γ and TNF-α in the coculture system (Fig. 5*B*), and elevated percentage of perforin^+^ and granzyme B^+^ cells (Fig. 5, *C* and *D*).

### ELF3 Promotes Transcriptional Activation of MUC16

**Figure 6.**
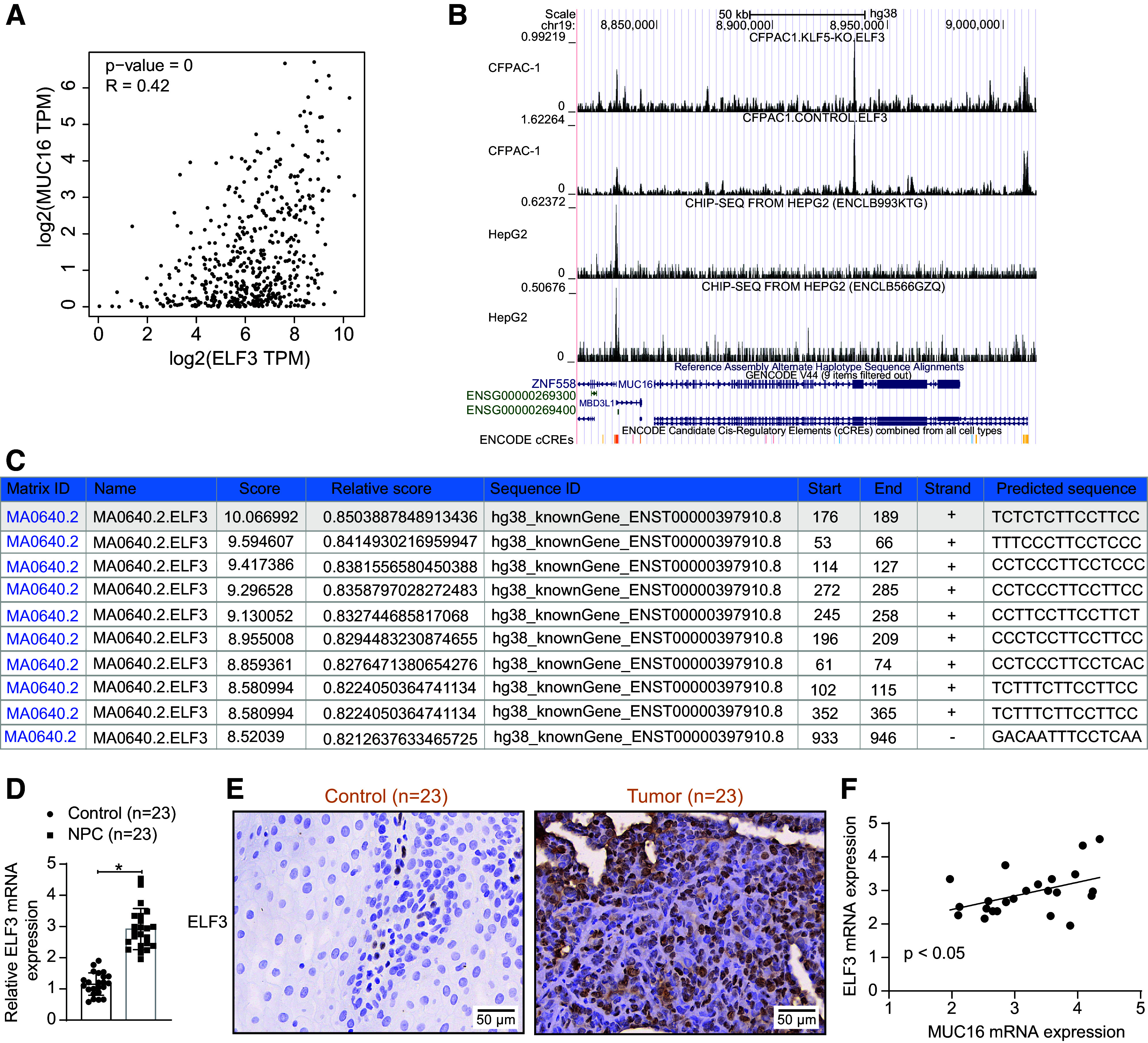
E74-like factor 3 (ELF3) is highly expressed in nasopharyngeal carcinoma (NPC). *A*: correlation between ELF3 and mucin-16 (MUC16) expression in head and neck squamous cell carcinoma (HNSC) analyzed by GEPIA database. *B*: Cistrome Data Browser database analysis of ELF3 binding at the MUC16 promoter. *C*: the binding site of ELF3 to the MUC16 promoter is predicted in the Jaspar database. *D*: ELF3 mRNA expression in NPC and adjacent normal tissues was analyzed using RT-qPCR (*n* = 23 patients). *E*: representative immunohistochemical staining images of ELF3 in NPC and adjacent normal tissues. *F*: the correlation between mRNA expression of ELF3 and MUC16 in NPC tissues (*n* = 23 patients) was analyzed using Pearson’s correlation coefficient. RT-qPCR, reverse transcription-quantitative polymerase chain reaction. *P < 0.05 (unpaired *t* test).

Despite revealing the downstream mechanism by which MUC16 promotes NPC progression, the reason for its high expression in NPC remains to be further analyzed. We focused on the presence of a unique transcription factor, ELF3, among the six DEGs at the intersection of the three GEO datasets. Analysis in GEPIA (http://gepia.cancer-pku.cn/index.html) informed that the expression of MUC16 in head and neck squamous cell carcinoma (HNSC) was positively correlated with ELF3 (Fig. 6*A*). Significantly binding peaks of ELF3 around the promoter of MUC16 were observed by ChIP-seq samples from the Cistrome Data Browser database (http://cistrome.org/db/#/) (Fig. 6*B*). In the Jaspar database, there are multiple ELF3 binding sites (Fig. 6*C*) on the MUC16 promoter fragment (chr19: 8981343–8982342).

First, the expression of ELF3 in NPC was analyzed, which demonstrated that ELF3 expression at both transcriptional and translational levels was elevated in NPC tissues (Fig. 6, *D* and *E*). Further correlation analysis showed that ELF3 mRNA and *MUC16* mRNA were significantly positively correlated in our cohort (Fig. 6*F*).

**Figure 7.**
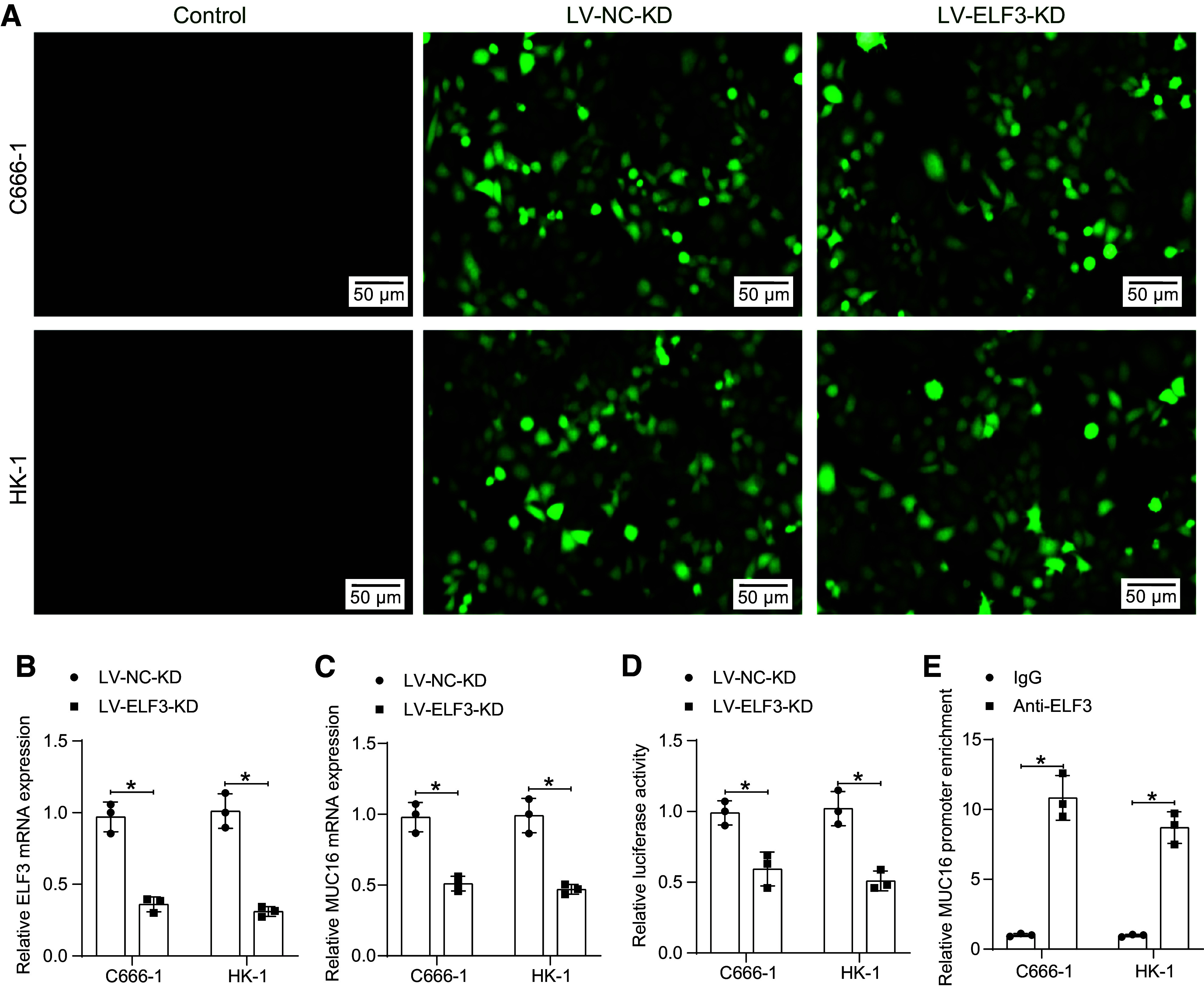
E74-like factor 3 (ELF3) mediates transcriptional activation of mucin-16 (MUC16). *A*: fluorescence microscopic observation of infection efficiency of ELF3 knockdown in C666-1 and HK-1 cells. ELF3 (*B*) and MUC16 (*C*) expression in C666-1 and HK-1 cells infected with lentiviral vectors harboring ELF3 knockdown were analyzed using RT-qPCR (*n* = 3). *D*: luciferase activity of luciferase vectors with MUC16 promoter sequence after knockdown of ELF3 in C666-1 and HK-1 cells was analyzed using a dual-luciferase reporter assay (*n* = 3). *E*: the enrichment of ELF3 at the MUC16 promoter in C666-1 and HK-1 cells was analyzed using chromatin immunoprecipitation (ChIP)-quantitative polymerase chain reaction (qPCR) (*n* = 3). Data are shown as mean values ± SD from three independent experiments. RT-qPCR, reverse transcription-quantitative polymerase chain reaction. *P < 0.05 (ANOVA).

Then, we knocked down the expression of ELF3 in C666-1 and HK-1 cells (Fig. 7, *A* and *B*) and analyzed the impact of ELF3 silencing on the expression of MUC16. The reduction of ELF3 downregulated MUC16 in NPC cells (Fig. 7*C*). Analysis of the effect of ELF3 on the transcriptional activity of MUC16 revealed that knockdown of ELF3 decreased luciferase activity, suggesting that the expression of ELF3 can promote the transcription of MUC16 (Fig. 7*D*). ChIP-qPCR experiments confirmed the presence of ELF3 binding at the MUC16 promoter, with significant enrichment of MUC16 in the DNA fragment pulled down by anti-ELF3 (Fig. 7*E*).

### ELF3 Activates MUC16 to Promote Glycolysis and Immune Escape in NPC Cells

**Figure 8.**
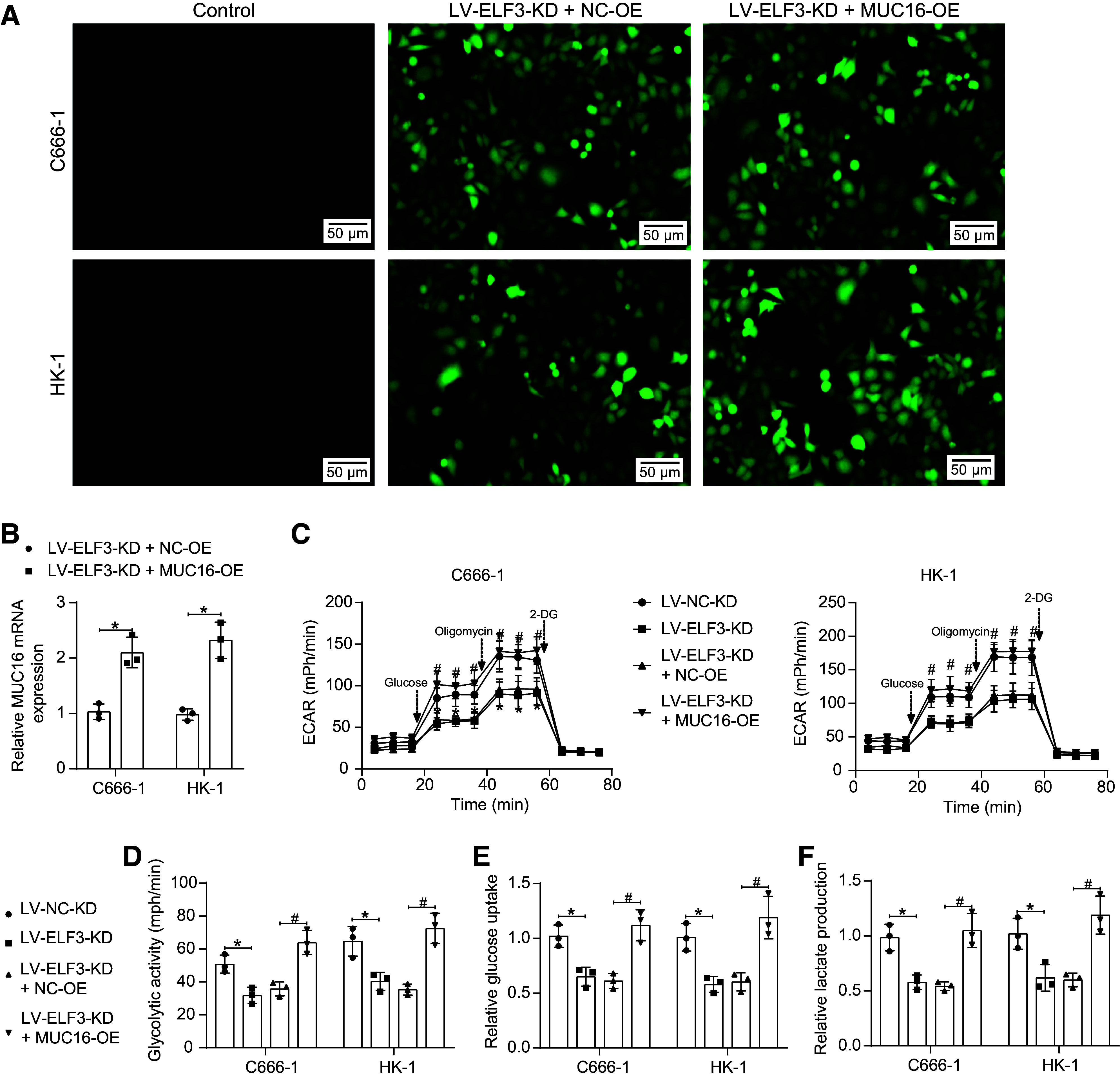
E74-like factor 3 (ELF3) activates mucin-16 (MUC16) to promote glycolysis in nasopharyngeal carcinoma (NPC). *A*: fluorescence microscopic observation of the infection efficiency of C666-1 and HK-1 cells (*n* = 3). *B*: detection of *MUC16* expression in C666-1 and HK-1 cells infected with lentiviral vector harboring ELF3 knockdown alone or in combination with *MUC16* analyzed using reverse transcription-quantitative polymerase chain reaction (RT-qPCR) (*n* = 3). *C*: assessment of extracellular acidification rate (ECAR) in C666-1 and HK-1 cells infected with lentiviral vectors (*n* = 3). *D*: glycolytic activity in C666-1 and HK-1 cells infected with lentiviral vectors (*n* = 3). *E*: glucose uptake capacity of C666-1 and HK-1 cells infected with lentiviral vectors (*n* = 3). *F*: lactate production of C666-1 and HK-1 cells infected with lentiviral vectors (*n* = 3). Data are shown as mean values ± SD from three independent experiments. *,#*P* < 0.05 (ANOVA).

Then, we overexpressed MUC16 in NPC cells with ELF3 knockdown. After verifying the overexpression efficiency (Fig. 8, *A* and *B*), we found that knocking down ELF3 decreased glycolytic activity, whereas overexpression of MUC16 increased glycolytic activity (Fig. 8, *C* and *D*). Notably, glucose uptake and lactate production measurements yielded similar results, and the knockdown of ELF3 impaired glucose uptake and reduced lactate production, which were abated by MUC16 upregulation (Fig. 8, *E* and *F*).

**Figure 9.**
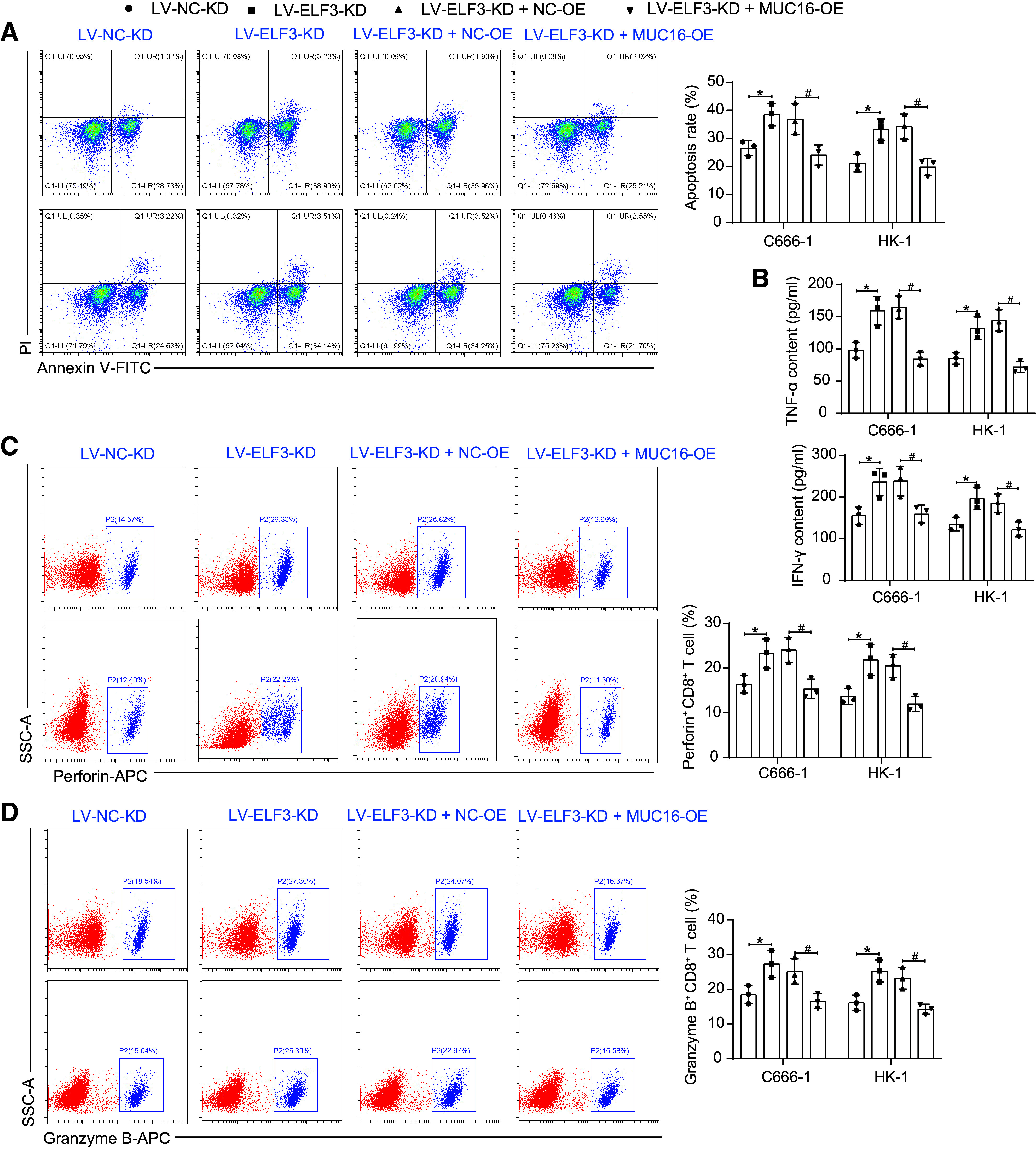
E74-like factor 3 (ELF3) activates mucin-16 (MUC16) to promote immune escape in nasopharyngeal carcinoma (NPC). *A*: detection of apoptosis in C666-1 and HK-1 cells after coculture of T cells with NPC cells by flow cytometry (*n* = 3). *B*: detection of TNF-α and IFN-γ in the coculture system of T cells and C666-1 and HK-1 cells was analyzed using ELISA (*n* = 3). Percentage of perforin^+^CD8^+^ T cells (*C*) and Granzyme B^+^CD8^+^ T cells (*D*) in T cells after coculture with C666-1 and HK-1 cells detected by flow cytometry (*n* = 3). Data are shown as mean values ± SD from three independent experiments. *,#*P* < 0.05 (ANOVA).

As for immune escape, after coculturing the NPC cells with T cells, knockdown of ELF3 induced NPC cell apoptosis (Fig. 9*A*), along with a promotion in the secretion of effectors by T cells in the coculture system (Fig. 9*B*), and a rise in the percentage of cytotoxic T cell activation (Fig. 9, *C* and *D*). By contrast, the overexpression of MUC16 led to T cell dysfunction, and T cell activation was blocked.

### ELF3 Activates MUC16 to Promote Tumor Growth in NPC

**Figure 10.**
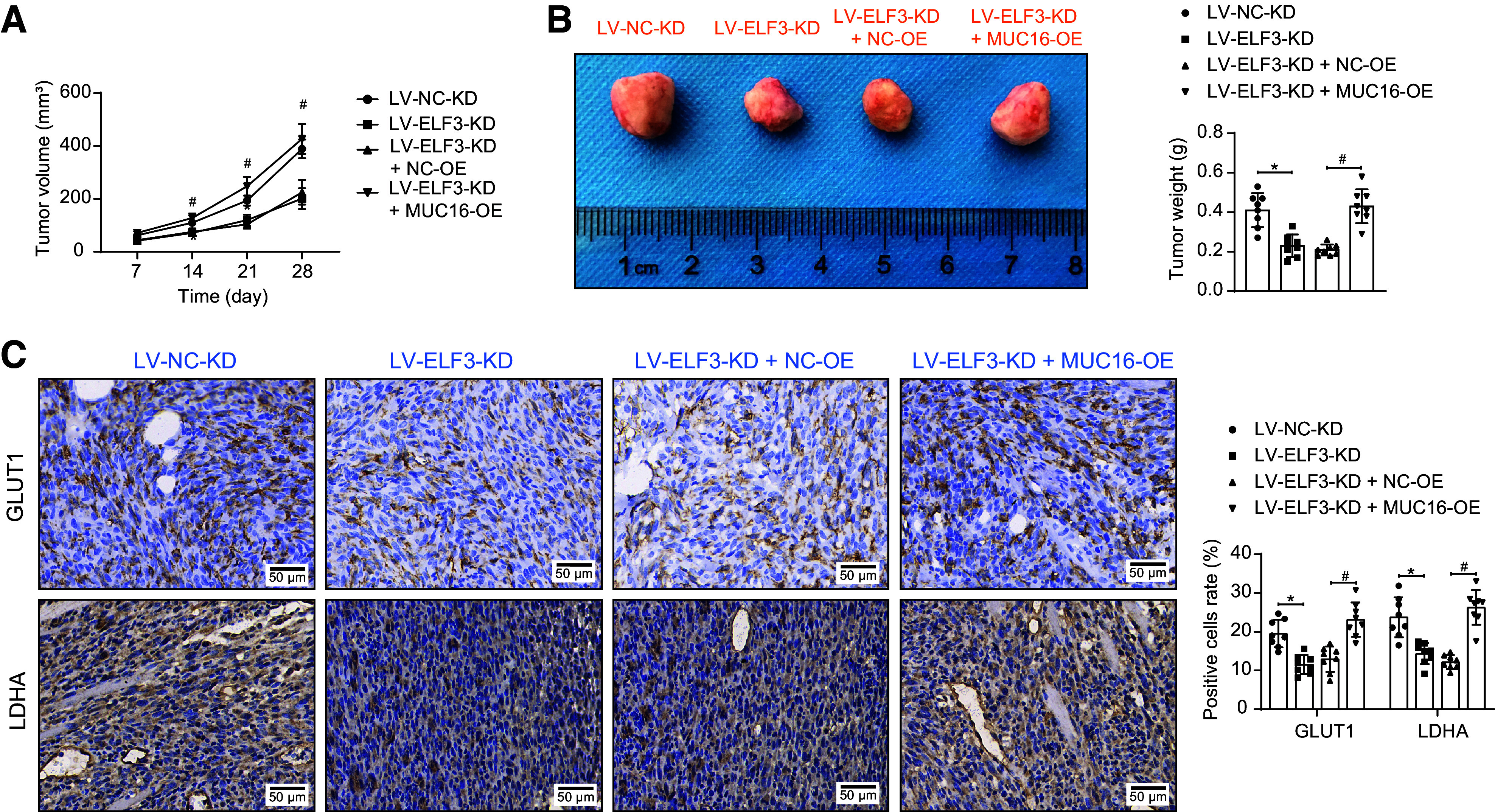
E74-like factor 3 (ELF3) activates mucin-16 (MUC16) to promote tumor growth in nasopharyngeal carcinoma (NPC). Subcutaneous xenograft growth curve (*A*) during the 28-day period and weight on the 28th day (*B*) after euthanasia of male mice (*n* = 8). *C*: expression of glucose transporter 1 (GLUT1) and LDHA in tumor tissues after knockdown of ELF3 and overexpression of MUC16 in male mice detected by immunohistochemistry (*n* = 8). Data are shown as mean values ± SD. *,#*P* < 0.05 (ANOVA).

We developed subcutaneous xenografts in mice using C666-1 cells. Knockdown of ELF3 in C666-1 cells slowed down tumor growth and reduced tumor burden, whereas overexpression of MUC16 promoted tumor growth instead (Fig. 10, *A* and *B*). Positive staining of GLUT1 and LDHA was reduced in tumor tissues formed by C666-1 cells with ELF3 knockdown. Overexpression of MUC16 reversed this effect (Fig. 10*C*).

**Figure 11.**
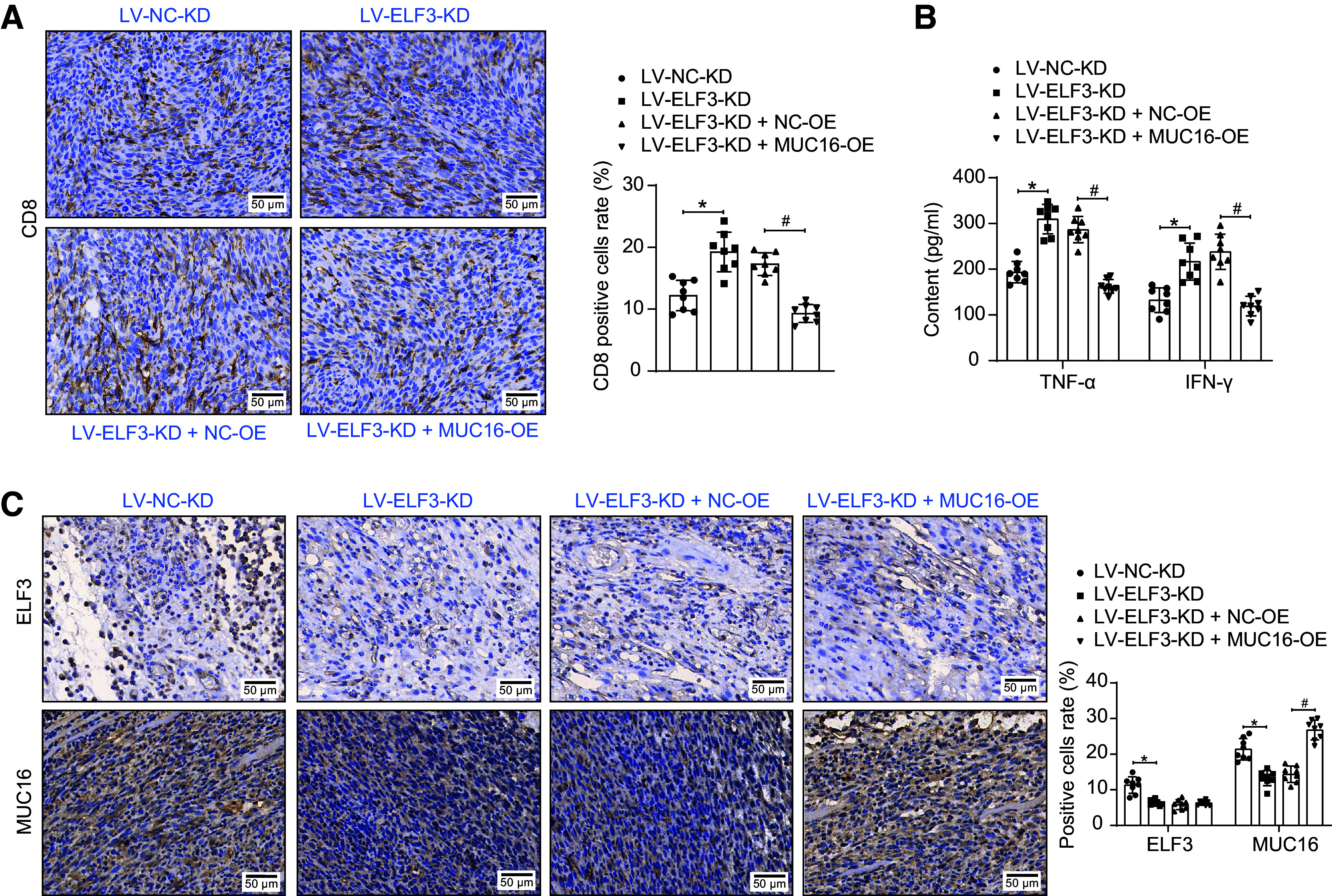
E74-like factor 3 (ELF3) activates mucin-16 (MUC16) to promote tumor growth in nasopharyngeal carcinoma (NPC) via immune escape. *A*: CD8^+^ T-cell infiltration in tumor tissues after knockdown of ELF3 and overexpression of MUC16 detected by immunohistochemistry (*n* = 8 mice/group). *B*: TNF-α and IFN-γ in tumor tissue homogenates after knockdown of ELF3 and overexpression of MUC16 analyzed using ELISA (*n* = 8 mice/group). *C*: ELF3 and MUC16 expression in tumor tissues after knockdown of ELF3 and overexpression of MUC16 detected by immunohistochemistry (*n* = 8). Data are shown as mean values ± SD. *,#*P* < 0.05 (ANOVA).

Assessment of T cell-mediated antitumor responses in tumor tissues showed that knockdown of ELF3 increased CD8^+^ T cell infiltration and elevated the levels of TNF-α and IFN-γ in tumor tissue homogenates. However, in tumor tissues formed by C666-1 cells with both ELF3-KD and MUC16-OE, an impaired antitumor response of T cells was observed (Fig. 11, *A* and *B*). Finally, the expression of ELF3 and MUC16 was decreased in the tumors of the ELF3-KD group, and the expression of MUC16 was increased after overexpression of MUC16 (Fig. 11*C*).

### Overexpression of ELF3 in NPC Is Associated with Its Reduced DNA Methylation Level

**Figure 12.**
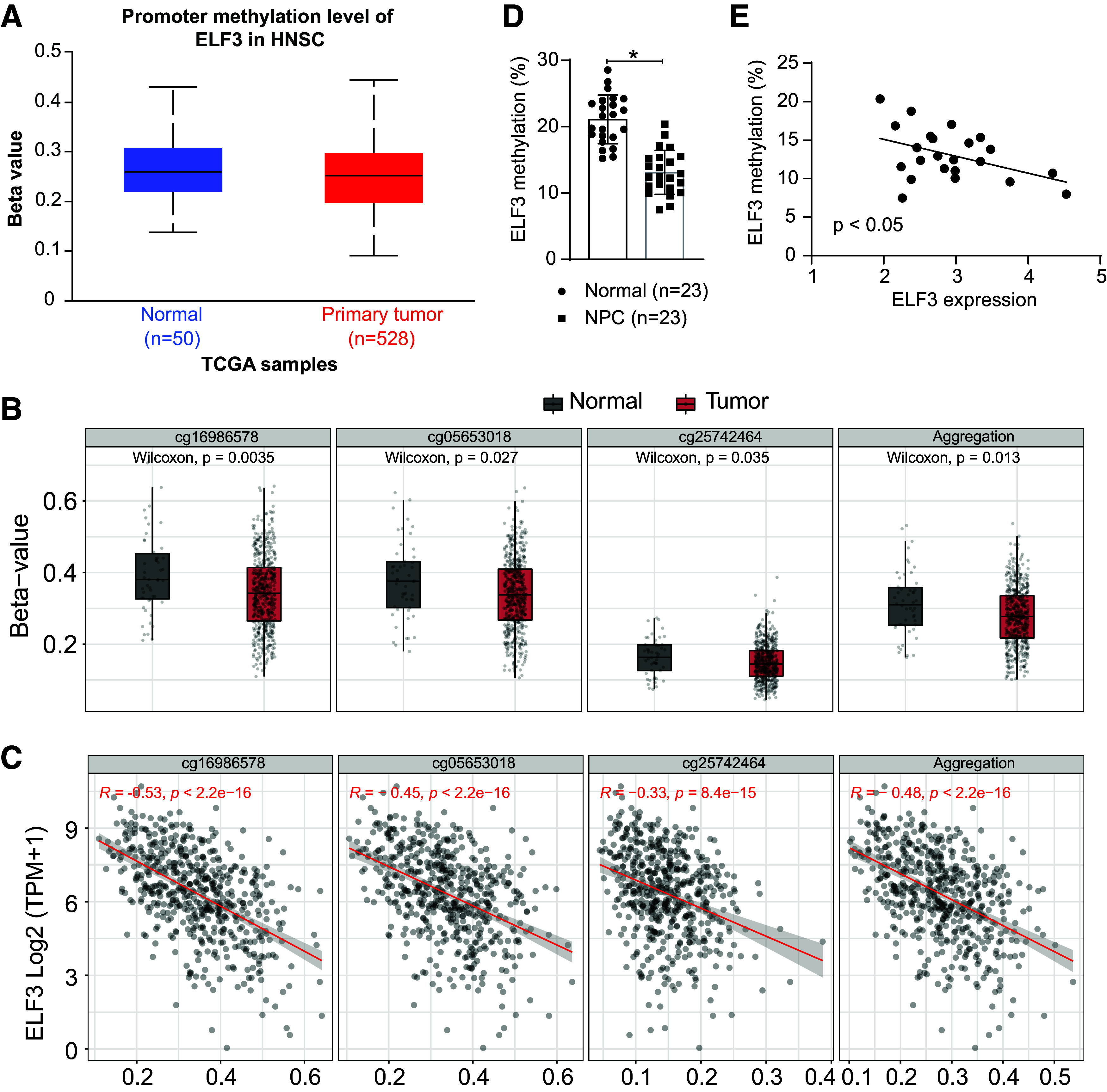
Overexpression of E74-like factor 3 (ELF3) in nasopharyngeal carcinoma (NPC) is associated with its reduced DNA methylation. *A*: methylation levels of ELF3 in head and neck squamous cell carcinoma (HNSC) predicted in the UALCAN database. *B*: specific CpG islands with reduced methylation in the ELF3 promoter are predicted on the Shiny Methylation Analysis Resource Tool (SMART) website. *C*: correlation between DNA methylation of ELF3 CpG islands and ELF3 expression in NPC. *D*: DNA methylation levels of ELF3 in tumor tissues of patients with NPC (*n* = 23 patients). *E*: the correlation between DNA methylation of ELF3 CpG islands and ELF3 expression in patients with NPC (*n* = 23 patients) was analyzed using Pearson’s correlation coefficient. Data are shown as mean values ± SD. *P < 0.05 (paired *t* test).

In the UALCAN database (https://ualcan.path.uab.edu/index.html), the total DNA methylation of ELF3 was reduced in HNSC (*P* = 4.298000*e*-02) (Fig. 12*A*). Specific CpG islands with reduced ELF3 promoter methylation in HNSC were analyzed by Shiny Methylation Analysis Resource Tool (SMART) (http://www.bioinfo-zs.com/smartapp/) (Fig. 12*B*). We observed that DNA methylation levels of these CpG islands were inversely correlated with ELF3 expression (Fig. 12*C*). Therefore, we hypothesized that the reduced level of DNA methylation is the direct cause of the overexpression of ELF3 in NPC.

First, we analyzed the DNA methylation of ELF3 in tumor tissues of patients with NPC using methylation-specific PCR. The methylation level of ELF3 in NPC tumor tissues was lower than that in control tissues (Fig. 12*D*). Its DNA methylation level was significantly negatively correlated with the expression of ELF3 (Fig. 12*E*).

**Figure 13.**
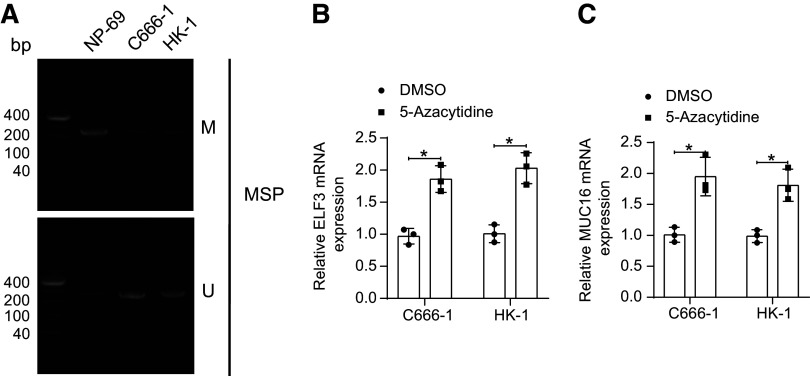
Decreased DNA methylation level of E74-like factor 3 (ELF3) induces its increased expression in nasopharyngeal carcinoma (NPC) cells. *A*: DNA methylation levels of ELF3 in nasopharyngeal epithelial NP-69 cells and NPC cells (C666-1 and HK-1 cells) were analyzed using methylation-specific PCR (MSP; *n* = 3). ELF3 (*B*) and mucin-16 (MUC16; *C*) expression in 5-azacytidine-treated C666-1 and HK-1 cells (*n* = 3). Data are shown as mean values ± SD from three independent experiments. *P < 0.05 (ANOVA).

Next, we analyzed the methylation of ELF3 in NPC cells and nasopharyngeal epithelial cells, and the methylation level of ELF3 was higher in the NPC cell line than in NP-69 cells (Fig. 13*A*). We then treated C666-1 and HK-1 cells using the DNA methylation inhibitor 5-azacytidine. Inhibition of DNA methylation prompted a significant increase in ELF3 expression (Fig. 13*B*) and MUC16 expression (Fig. 13*C*).

## DISCUSSION

**Figure 14.**
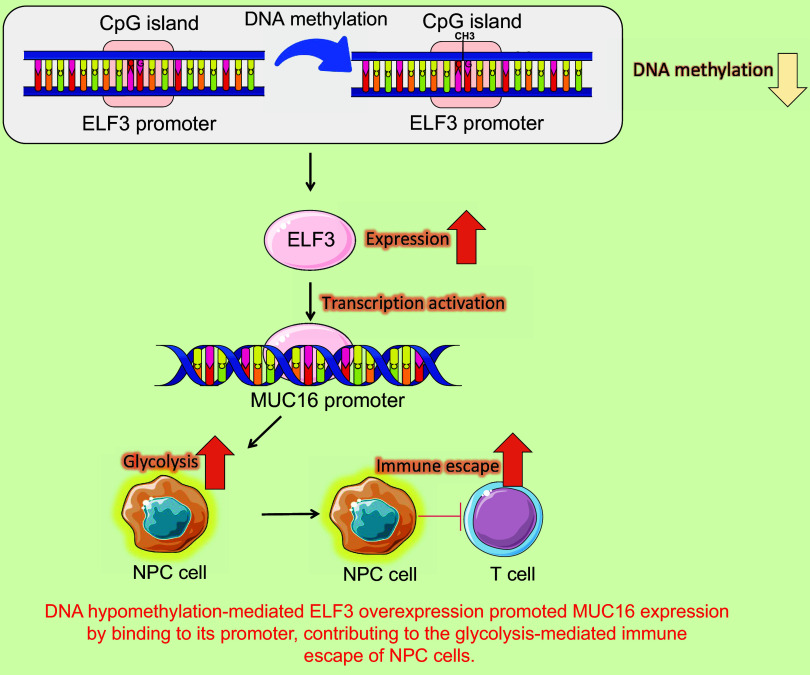
Working model for regulation of nasopharyngeal carcinoma (NPC) progression by ELF3/MUC16 axis. Figure contains images from Servier Medical Art (https://smart.servier.com) licensed under a CC BY 4.0 license (https://creativecommons.org/licenses/by/4.0/). ELF3, E74-like factor 3; MUC16, mucin-16.

The present study uncovered new roles of MUC16 in promoting glycolysis-related immune escape of NPC cells. Our results demonstrate that elevated MUC16 expression was tightly linked to the TNM stage and recurrence of patients with NPC. High transcription of MUC16 induced by ELF3 could induce aerobic glycolysis to promote NPC cell escape from T cells. Finally, the overexpression of ELF3 in NPC was related to the hypomethylation of its promoter (Fig. 14).

Saad et al. (16) summarized that as a screening test for lung and ovarian cancer diagnosis and prognosis in the early stages, MUC16 has been used as a marker in three different clinical settings. Here, we identified a remarkable correlation between MUC16 high expression and TNM stage and recurrence of patients with NPC, indicating the possible diagnostic role of MUC16 in NPC. Marimuthu et al. (17) found that MUC16 knockdown in pancreatic ductal adenocarcinoma cells significantly decreased colony formation and migration. By using lentiviral vectors, we silenced MUC16 cells in NPC cells and observed similar suppressing effects of MUC16-KD on NPC cell viability and mobility. MUC16 can manipulate glucose uptake by controlling GLUT1 in epithelial ovarian cancer cells, thus promoting glycogen synthesis, so that tumor cells produce more energy for proliferation (18). Here, we also observed the repressed glycolysis of NPC cells in response to LV-MUC16-KD treatment, as manifested by reduced ECAR, glucose uptake, lactate production, and expression of glycolysis-related proteins. Besides MUC16, other members of the mucin family, including MUC19 and MUC1 have been implicated in the glucose metabolism of different cancers, including breast cancer (19), bladder cancer (20), and endometrial carcinoma (21). However, the detailed mechanisms for these extracellular glycoproteins regulating glycolysis remain unclear and await further exploration.

It has been revealed that enhanced levels of extracellular lactate inhibited cytotoxic T cell activity, resulting in the invasive characteristic of lung cancer cells (22). Considering that MUC16 has been linked to the immune response in patients with gastric cancer (23), we anticipated that MUC16 induced glycolysis, thereby helping the NPC cells escape from immune surveillance. In NPC, T cells are the main component of the TME, and these T cells are recruited by chemokines that are produced by the tumor cells (23). Here, the proportion of cytotoxic T cells and the production of the released cytokines were found to be enhanced in response to MUC16 knockdown in NPC cells. 2-DG (a glucose analog and an inhibitor of glucose metabolism) has been reported to block the promotion of NPC cell survival and proliferation (24). Also, the 2-DG treatment of NPC cells enhanced the effector function of cytotoxic T cells.

Subsequently, we identified the binding relation between ELF3 and the MUC16 promoter. Consistently, Enfield et al. (25) revealed that ELF3 displayed strong prognostic value in lung adenocarcinoma, and ELF3 expression was required for tumor growth. More specifically, the expression of ELF3 was associated with metastasis and TNM stage in patients with NPC (26). Silencing of ELF3 with siRNA repressed the growth, clone formation, migration, and invasion of thyroid cancer cells by regulating the transcription of the human epidermal growth factor receptor 2 family of receptors (27). However, its role as a transcription factor in NPC has not been explored. Conditioned medium from biliary tract cancer cells overexpressing ELF3 enhanced the migration of natural killer cells and CD8^+^ T cells toward the conditioned medium (28). Moreover, a majority of ELF3 alterations are frame-shift mutations that contribute to cancer-specific neoantigens that activate T-cells, which indicates that they are cancer vaccine candidates (29). The loss-of-function assays performed here revealed that the ELF3 downregulation repressed the glycolysis and immune escape of NPC cells, which were reversed by MUC16 overexpression. These in vitro findings were reproduced in vivo. Finally, we attributed the high expression of ELF3 to the hypomethylation of its CpG island. High methylation in cg07154254 (ELF3) was related to longer overall survival in patients with thymic epithelial tumors (30). Here, the mRNA expression of ELF3 and MUC16 was enhanced following DNA methylation inhibitor 5-azacytidine. Further studies are warranted to identify the upstream modifiers that can affect the DNA methylation of ELF3 in NPC.

All in all, we corroborated the functions of ELF3 in glycolysis and the immune escape of NPC cells. As a transcription factor, ELF3 promoted MUC16 expression by binding to its promoter, contributing to the glycolysis-mediated immune escape of NPC cells. The high expression of ELF3 was related to the DNA hypomethylation. Targeting the ELF3/MUC16 axis generates a superior antitumor immune response, which will help establish an innovative approach to restore protective antitumor immunity for NPC immunotherapy.

## DATA AVAILABILITY

The datasets used and/or analyzed during the current study are available from the corresponding author upon reasonable request.

## DISCLOSURES

No conflicts of interest, financial or otherwise, are declared by the authors.

## AUTHOR CONTRIBUTIONS

Y.L., H.Z., Q.Y., and Q.W. conceived and designed research; Y.L. and H.Z. performed experiments; Y.L., H.Z., Q.Y., and Q.W. analyzed data; Y.L. prepared figures; H.Z., Q.Y., and Q.W. drafted manuscript; Y.L., H.Z., Q.Y., and Q.W. edited and revised manuscript; Y.L., H.Z., Q.Y., and Q.W. approved final version of manuscript.
